# ﻿Redescription of *Apocorophiumacutum* (Crustacea, Amphipoda, Corophiidae) with material from type locality and key of world *Apocorophium* species

**DOI:** 10.3897/zookeys.1106.83340

**Published:** 2022-06-17

**Authors:** Benoit Gouillieux, Hugues Blanchet, Patrice Gonzalez

**Affiliations:** 1 Université de Bordeaux, CNRS, EPOC, EPHE, UMR 5805, F-33600 Pessac, France Université de Bordeaux Pessac France

**Keywords:** Corophiini, ecology, redescription, world key

## Abstract

*Apocorophiumacutum* (Chevreux, 1908), the type species of the genus, was originally but only partially described by Chevreux with female specimens from Bônes (Algeria); male specimens were later described from Brittany (France). Since then, the species has been recorded in different places of the world, some of them questionable. Herein, the species is entirely redescribed with material from the type locality and Brittany, and additional material from Arcachon Bay is studied to provide biological data. The known geographical distribution of this species is summarized, and a world identification key of *Apocorophium* species is also given.

## ﻿Introduction

Corophiini Leach, 1814 are generally tube-dwelling amphipods present in various marine, estuarine, and freshwater habitats, including in sandy to muddy bottoms, with hydrozoa, on algae, and among oysters, and some species can be commensal (Crawford 1937; Lincoln 1979; Bousfield and Hoover 1997). Some genera and species have a worldwide distribution. Bousfield and Hoover (1997) reviewed in depth the superfamily Corophioidea Leach, 1814 and described the subfamily Corophiinae, 12 new genera, and a new species. Later, Myers and Lowry (2003) provided a new classification, dividing the suborder Corophiidea Leach, 1814 into two infraorders (Corophiida and Caprellida Leach, 1814) and reassigned the authority of Corophiini, Corophiinae, Corophiidae, and Corophiida to Leach (1814). Lowry and Myers (2013) replaced Corophiidea by Senticaudata Lowry & Myers, 2013.

The identification key by Bousfield and Hoover (1997) is widely used around the world, but unfortunately, the study of some original descriptions of selected Corophiini species show that their key contains mistakes; this was rectified by Gouillieux and Sauriau (2019) in their key to the species characterized by urosome segments fused with uropod 1 arising mainly ventrally.

According to Horton et al. (2021), five species belong to the genus *Apocorophium*: *A.acutum* (Chevreux, 1908), *A.curumim* Valério-Berardo & Thiago de Souza, 2009, *A.lacustre* (Vanhöffen, 1911), *A.louisianum* (Shoemaker, 1934), and *A.simile* (Shoemaker, 1934). *Apocorophiumacutum*, the type species of the genus, was partially described by Chevreux (1908) and Chevreux and Fage (1925) from the Algerian Mediterranean and French Atlantic coasts. It is a well-known Mediterranean and Atlantic species (Crawford 1937; Myers 1982; Bachelet et al. 2003) and was subsequently recorded in different areas around the world with more or less detailed descriptions; however, some of these are doubtful. This paper provides a complete redescription of *A.acutum* based on females specimens from Bône (the type locality) and male specimens from Brittany (the geographical area of the first description of a male specimen), additional ecological and biological information, and an identification key to the world *Apocorophium* species.

## ﻿Materials and methods

**Figure 1.**
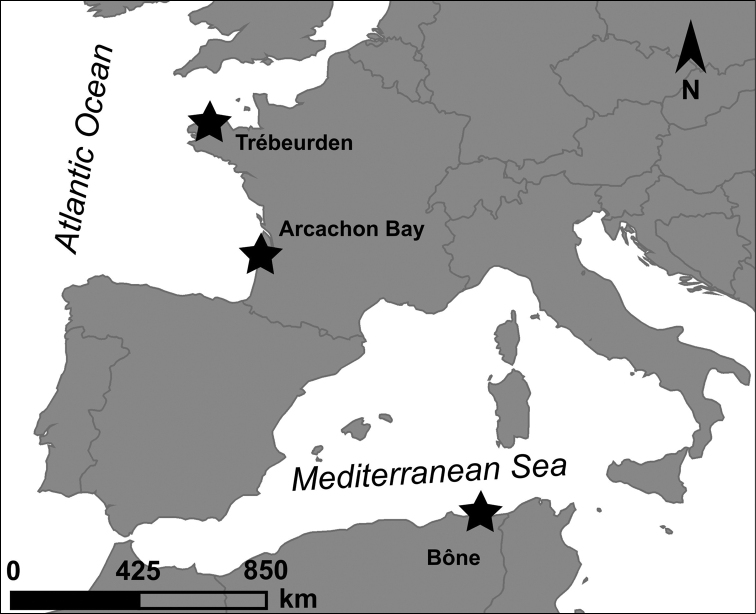
Sampling stations of *Apocorophiumacutum* (Chevreux, 1908) used in this study. Bône (Algeria): female locality type; Trébeurden (France): 5 km from the location of the first description of a male; Arcachon Bay (France): additional material examined.

Specimens of *Apocorophiumacutum* examined come from three different localities (Fig. 1): (1) Bône, Algeria, type locality of female specimens; (2) Trébeurden, Brittany, France, 5 km from Lanion River, which is the geographical area of the first description of male specimens, and (3) Arcachon Bay, France. The specimens were examined with a stereomicroscope and a compound microscope. Body length (BL) from the anterior margin of head to the posterior end of telson and eggs’ size were measured with NIS-Elements Analysis software. Females from Arcachon Bay (stations “bouée 13” and “Arcachon harbor”) with intact brood pouches were separated in order to measure their fecundity: the eggs were removed from the brood pouch of each female, counted, and their diameter measured. Specimens from a third location in Arcachon Bay (station “blockhaus”) were used to evaluate the relationship between body length and gender features (male characteristics features, oostegite shape and presence of eggs). Relationships between quantitative variables such as body length in females and number of eggs in the marsupium were assessed by Spearman’s rank correlation in order to potentially show a possible non-linear but monotone relationship between variables (Hollander and Wolfe 1973). Differences in body length among morphologically different specimens (e.g., males, egg-bearing female, female with smooth oostegite) were tested using non-parametric Kruskal-Wallis test followed by post-hoc pairwise Dunn test where significant differences were assessed by Kruskal-Wallis test (Hollander and Wolfe 1973).

For scanning electron microscope (SEM) studies, specimens were dehydrated in a graded ethanol series, critical-point dried, sputter coated with gold, and examined with a scanning electron microscope. Drawings were carried out from pictures using INKSCAPE software (v. 0.92). Specimens from Bône were loaned from the Muséum national d’Histoire naturelle (MNHN, Paris), and some specimens studied from Trébeurden were deposited in the MNHN, Paris. Molecular analyses on specimens from Trébeurden and Arcachon were carried out and sequences are available in GenBank (GenBank accession numbers: ON455206 to ON455209). Unfortunately, it was not possible to do molecular analyses with material from the type locality to compare with French specimens.

## ﻿Results

### ﻿Systematics

**Order Amphipoda Latreille, 1816**

**Suborder Senticaudata Lowry & Myers, 2013**

**Family Corophiidae Leach, 1814**

**Subfamily Corophiinae Leach, 1814**

**Tribe Corophiini Leach, 1814**

#### Genus *Apocorophium* Bousfield & Hoover, 1997

##### ﻿ Apocorophium acutum

Taxon classificationAnimaliaAmphipodaCorophiidae

(Chevreux, 1908)

97590B10-F1C0-54ED-B993-47EF1973334C

**Figure 2.**
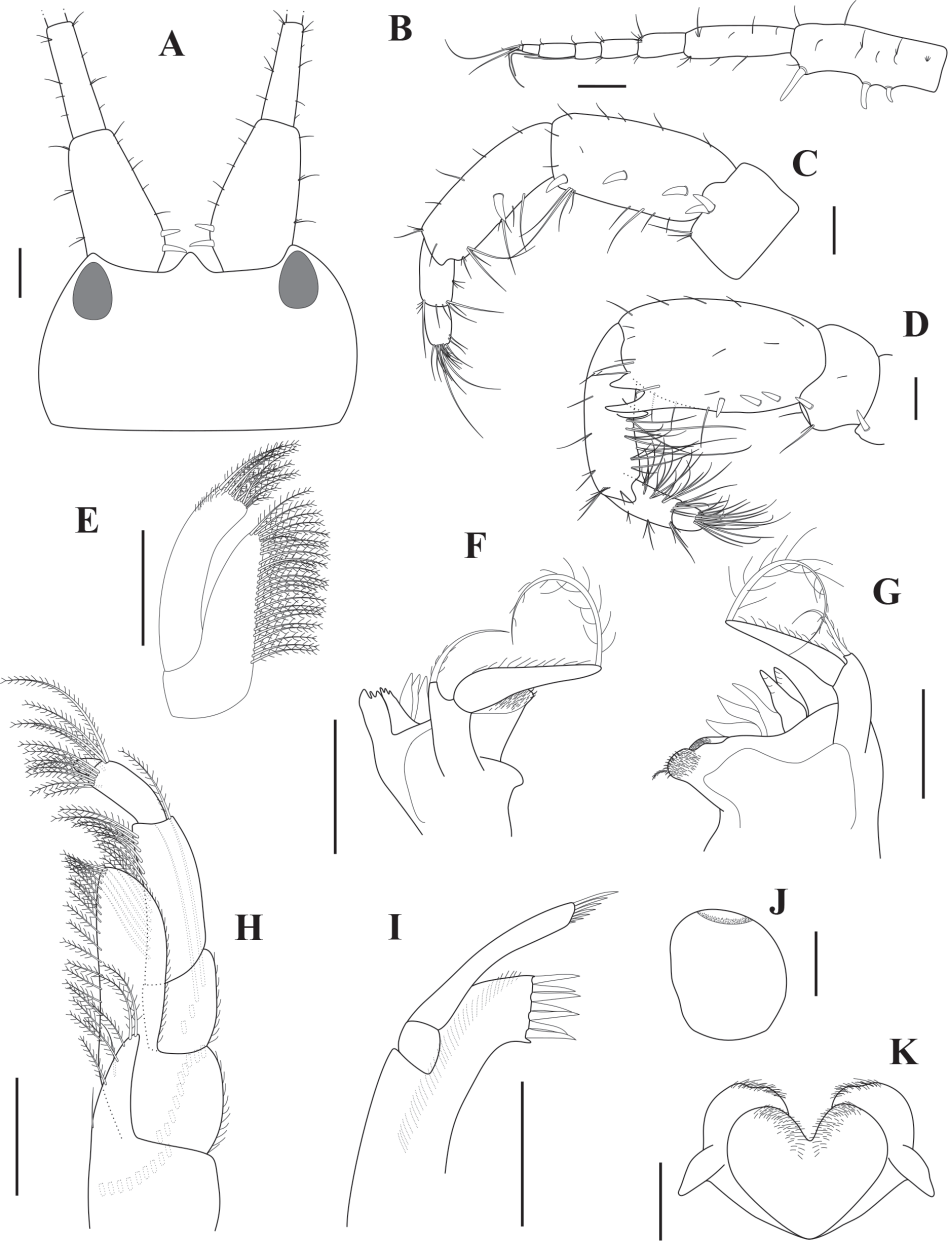
*Apocorophiumacutum* (Chevreux, 1908). (**A–C**) brooding female, BL = 3.19 mm, MNHN-IU-2013-19982, Bône (**D**) male, BL = 2.32 mm, MNHN-IU-2016-3392, Trébeurden (**E–K**) female, BL = 3.55 mm, MNHN-IU-2016-3401, Bône. **A** head and antenna 1, dorsal view **B** left antenna 1, outer view **C** right female antenna 2, inner view **D** right male antenna 2, inner view **E** maxilla 2 **F** left mandible **G** right mandible **H** maxilliped **I** maxilla 1 **J** upper lip **K** lower lip. Scale bars: 0.1 mm.

**Figure 3.**
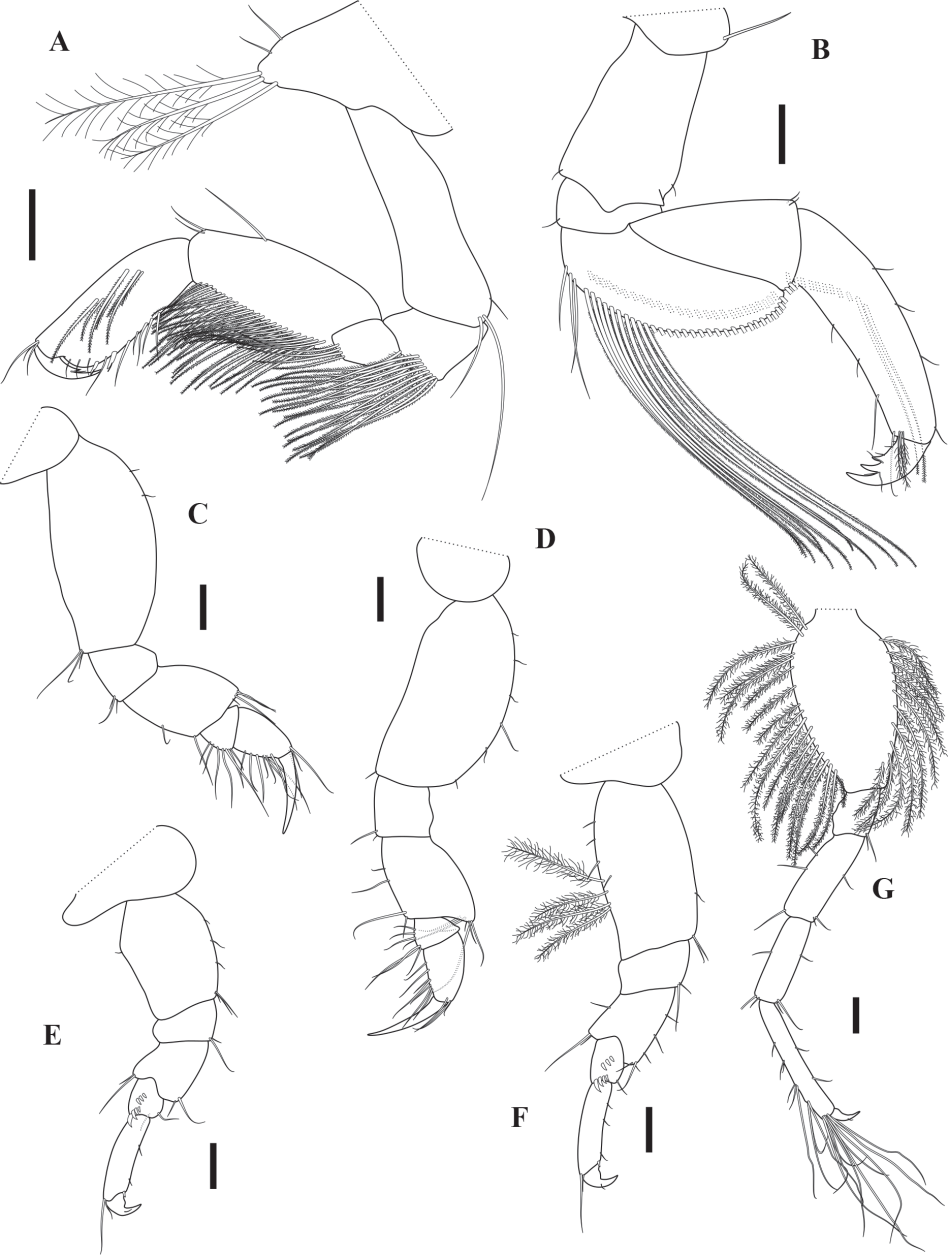
*Apocorophiumacutum* (Chevreux, 1908), female BL = 3.55 mm, MNHN-IU-2016-3401. **A** right gnathopod 1, inner view **B** right gnathopod 2, outer view **C–G** right pereopods 3–7, outer view. Scale bars: 0.1 mm.

Corophium acutum Chevreux 1908: 75 (original description), fig. 6.—1910: 271, h.—Chevreux and Fage 1925: 366–367, figs 359, 374.—Poisson and Legueux 1926: 320–325, figs 5,6 (Banyuls form).—Schellenberg 1928: 672–673.—Shoemaker 1934: 26–27.—Crawford 1937: 624–625.—Ruffo 1938: 147–148.—Salfi 1939: 31–62, figs 1–7.—Shoemaker 1947: 59, fig. 9.—Hurley 1954: 439–442, figs 2, 3 (in part).—Barnard 1969: 42.—Bousfield 1973: 205, fig. LXIV.—Lincoln 1979: 534, fig. 256 (in part).— Dickinson et al. 1980: 12.—Myers 1982 (in Ruffo ed.): male 188–190, fig. 126.—Barnard and Karaman 1991: 185.
Apocorophium acutum Bousfield and Hoover 1997: 123–125, fig. 35.— Lowry and Stoddart 2003: 88.— Ren 2006: 296–297, fig. 117.— Bano and Kazmi 2012: 113.—Hossain and Hughes 2016: 376–381, figs 2–5.
Not CorophiumacutumPoisson and Legueux 1926: 320–325, figs 5, 6 (Caen form).—Myers 1982 (in Ruffo ed.): female 188–190, fig. 126.
Doubtful ApocorophiumacutumJung and Kim 2007: 247–250, fig. 1.— Demicheli and Verdi: 2018, 1169–1173, figs 1–3.

###### Material examined.

Algeria • 6 brooding females and 1 juvenile; Bône (type locality); 4 May 1900; MNHN-IU-2013-19982; campaign MELITA, St. 677 “Melita II”, on concrete blocks removed from the harbor • 1 female, same data as for preceding; MNHN-IU-2016-3401; dissected brooding specimen.

France • many males and females; Arcachon Bay / station “bouée 13”; 44°38'07.20"N, 001°14'06.60"W; 2 m depth; 20 September 2014; Benoit Gouillieux leg.; mussels, hand-collected on submerged part of a navigation buoy; MNHN-IU-2016-3426 and MNHN-IU-2016-3427 • many males and females; Arcachon Bay / station “Arcachon harbor”; 44°39'36.53"N, 001°09'06.59"W; 0.5 m depth; 20 February 2020; Benoit Gouillieux leg.; on floating pontoons in harbor • 414 specimens; Arcachon Bay / station “blockhaus”; 44°34'00.40"N, 001°14'15.14"W; 5 m depth; between May 2018 and April 2019; Benoit Gouillieux leg.; with Hydrozoa*Amphisbetiaoperculata* (Linnaeus, 1758) • many males and females; Trébeurden / harbor; 48°46'12.21"N, 003°35'09.71"W; 0.5 m depth; 1 February 2020; Gabin Droual leg.; on floating pontoons (Port); MNHN-IU-2016-3392.

###### Description

**Figure 4.**
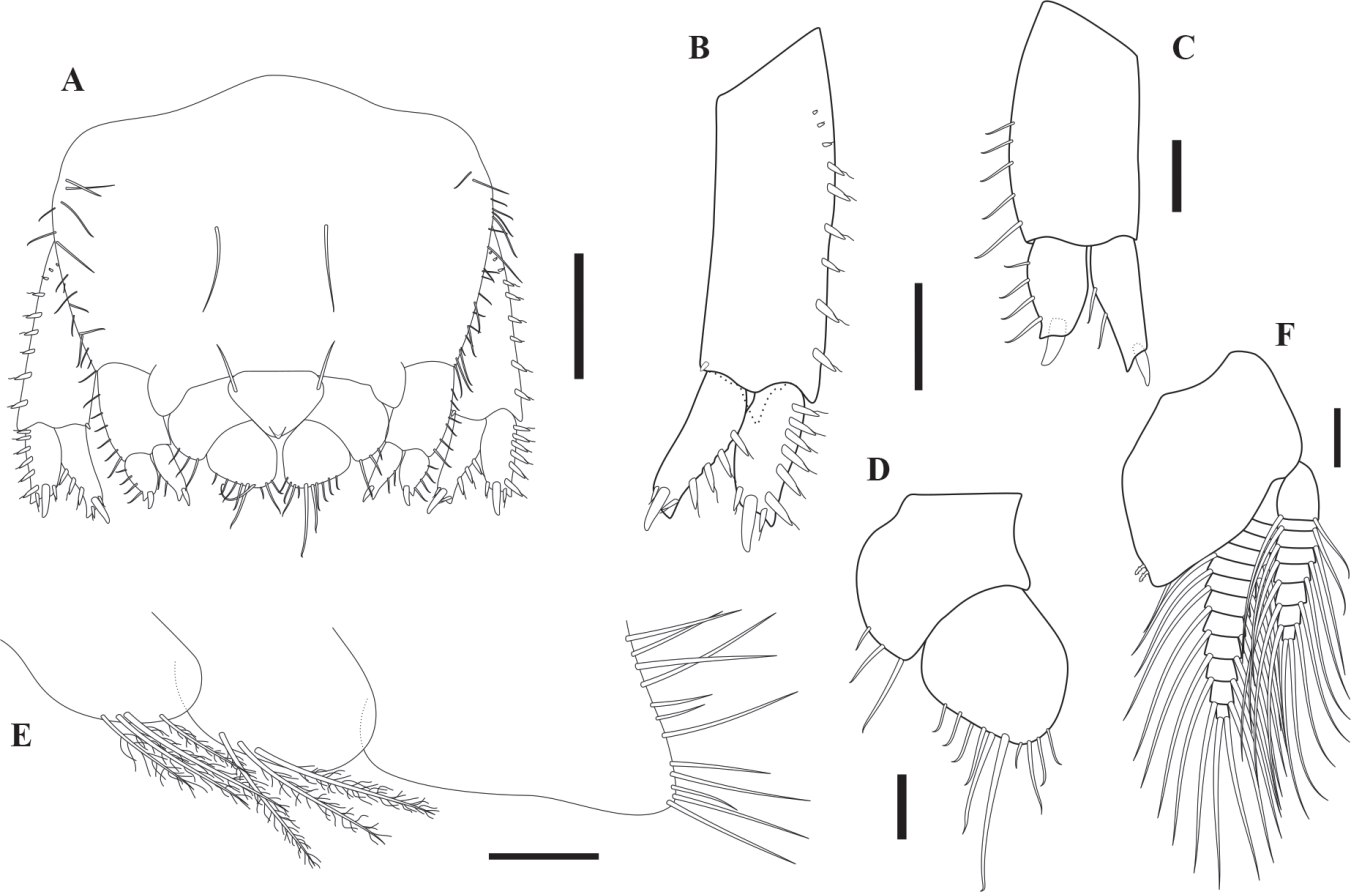
*Apocorophiumacutum* (Chevreux, 1908), female BL = 3.55 mm, MNHN-IU-2016-3401 **A** pleotelson, dorsal view **B** uropod 1, dorsal view **C** uropod 2, ventral view **D** uropod 3, dorsal view **E** right epimeral plates 1–3, outer view **F** pleopod 1, dorsal view. Scale bars: 0.2 mm (**A**); 0.1 mm (**B, D–F**); 0.05 mm (**C**).

**Figure 5.**
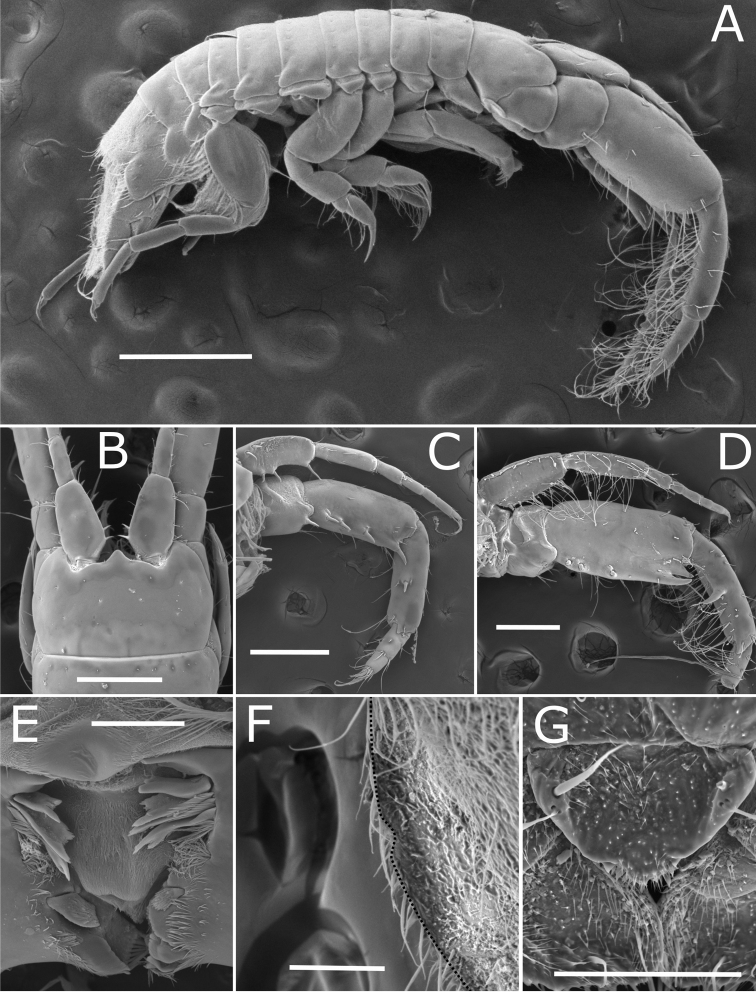
*Apocorophiumacutum* (Chevreux, 1908), specimens from Arcachon Bay, station “bouée 13”, France, 20/09/2014 **A** male specimen, lateral view **B** female head, dorsal view **C** female antenna 1 and 2, inner view **D** male antenna 1 and 2, inner view **E** mandibles **F** female pleotelson, dorsolateral view showing vestigial notch (dotted edge) **G** female, telson, dorsal view. Scale bars: 0.5 mm (**A**); 0.250 mm (**B–D**); 0.05 mm (**E**); 0.1 mm (**F, G**).

**(Figs 2–5).** Based on adults females, Chevreux collection, MNHN-IU-2016-3401 and MNHN-IU-2013-19982, Bône, Algeria, 4 May 1900, campaign MELITA, St. 677 “Melita II”, type locality.

**Head. *Head*** with rostrum pointed distally, triangular in dorsal view, reaching lateral ridge of head. ***Eyes*** visible in alcohol. ***Antenna 1*** weakly setose; peduncular article 1 rectangular, ventral margin with three robust setae, dorsomedial margin with two robust setae; length ratio of peduncular articles 1–3 = 1.00 : 0.72 : 0.31; flagellum 5-articulate, shorter than peduncle, articles 2–4 with a small aesthetascs ventrodistally. ***Antenna 2*** peduncular article 3 wider than long, with a pair of mediodistal robust setae; peduncular article 4 with three solitary robust setae; peduncular article 5 with a robust seta medially and a small process mediodistally; flagellum 3-articulate, distal article tiny with two robust setae. ***Lower lip*** inner lobe subovate, coalescent proximally, rounded apically; mandibular process small and blunt; both lobes covered with patch of pubescence medially. ***Right mandible*** well developed, incisor process and lacinia mobilis produced inward, bluntly tridentate; accessory setal row with three curved, finely pectinate blades, followed by tuft of pappose setae and a brush-like seta; molar well developed, massive, truncate; palp biarticulate, proximal segment shorter than distal, with 1 finely plumose seta apically, distal segment slender, with pubescence medially and long plumose seta apically. ***Left mandible*** similar, except for molar process which presents 2 additional blades. ***Maxilla 1*** outer plate armed with seven setal-teeth apically; palp biarticulate, proximal segment short, distal one extending beyond end of outer lobe, with row of seven distal setae. ***Maxilla 2*** inner plate with longitudinal row of pinnate setae on inner and distal margins; outer plate extending beyond end of inner one, with row of pinnate setae on distal margin. ***Maxilliped*** inner plate slender and elongate, basal portion with row of about nine plumose setae, inner margin with four and two pinnate setae; outer plate not reaching distal end of palp article 2, basal portion with row of about ten plumose setae, inner margin densely setose; palp 4-articulate, article 2 elongate, about three times as long as wide, inner margin densely setose, outer margin with one plumose seta distally, article 3 with rounded distal corner, distal article small, 0.24× article 3, with apical setae.

***Pereon*. *Gnathopod 1*** subchelate; coxa ventral margin with three long plumose setae, anterior margin with two simple setae; basis anterior margin unarmed, posterodistal corner with unequal setae; ischium quadrate, with long pinnate setae posterodistally; merus short, with long pinnate setae posterodistally; carpus slightly narrowing distally, anterior margin with one median and two distal simple setae, posterior margin with two rows of pinnate setae; propodus 0.9× carpus, posterior margin slightly convex, medial portion with pectinate setae, palm transverse, slightly convex, edge laminar and transversally striated, limited posteriorly by two robust setae; dactylus falcate. ***Gnathopod 2*** simple; coxa small, with one long simple seta anteriorly; basis subrectangular, anterodistal and posterodistal corners with a simple seta; ischium flat, depressed, posterodistal corner with a simple seta; merus convexly curved posteriorly, with two rows of long pinnate setae along posterior margin and medial portion; carpus isosceles triangle in shape, strongly widening distally, with two small simple and few long pinnate setae posterodistally; propodus weakly narrowing distally, 1.6× carpus, proximal third of medial portion with oblique row of pinnate setae, anterior and posterior margins sparsely setose, posterodistal corner with simple and plumose setae; dactylus short, flexor margin with two teeth and simple setae.

***Pereopod 3*** coxa small; basis weakly expanded medially, anterior margin with two setules, posterior margin bare, posterodistal corner with cluster of simple setae; merus anterodistal corner with cluster of simple setae, posterior margin with one medial and one distal simple seta; carpus small, subtriangular, with setae on anterior margin; propodus about twice as long as carpus, posterior margin and anterodistal corner with simple setae; dactylus simple, subequal in length to carpus and propodus length together. ***Pereopod 4*** similar to pereopod 3, except basis anterior margin slightly more setose. ***Pereopod 5*** coxa depressed, much wider than long, narrowing distally; basis slightly widened medially, anterior margin weakly setose, posterior margin with one setule; merus widening distally, antero and posterodistal corners with simple setae; carpus short, with two oblique rows of three proximal and four distal robust setae respectively; propodus about four times as long as wide, weakly setose; dactylus short. ***Pereopod 6*** similar to pereopod 5, but about 1.3× longer; basis more subrectangular, with a row of setules and four plumose setae. ***Pereopod 7*** elongate, much longer than either pereopod 5 or 6; basis elongate-ovate, moderately expanded anteriorly, densely setose along both margins with long plumose setae; ischium to propodus linear and rectangular; length ratio of articles 2–7 = 1.00 : 0.26 : 0.52 : 0.5 : 0.56 : 0.16.

**Pleon. *Epimera 1–2*** subovate, ventral margins rounded, with long plumose setae; ***epimeron 3*** subrectangular, distinctly longer than epimera 1–2, ventral margin bare, hind margin with many long simple setae. ***Urosomites 1–3*** fused, without notch laterally; uropod 1 arising mainly ventrally. ***Uropod 1*** peduncle rectangular, about 2.2× outer ramus, ventrodistal process present, triangular, blunt, lateral margin with row of robust setae, proximal ones short and setae like, medial margin bare except a small distal robust seta; outer ramus slightly shorter than inner, lateral margin with six robust setae, medial margin bare, three subdistal robust setae, the middle one the longest; inner ramus slightly curved medially, lateral margin with four robust setae, medial margin bare, three subdistal robust setae, the middle one the longest. ***Uropod 2*** peduncle longer than rami, without ventrodistal process, outer margin with setae on distal half; rami with one distal robust seta; outer ramus slightly shorter than inner with simple setae marginally. ***Uropod 3*** uniramous, peduncle short, broad, with three simple setae on outer margin; ramus subelliptical, narrowing distally, margins with unequal simple setae. ***Telson*** fleshy, thickened, grooved centrally, subtriangular, broadest in middle.

***Male*** (sexually dimorphic characters, based on specimens from Trébeurden, France, 1 February 2020, MNHN-IU-2016-3392).

***Antenna 1 and 2*** (Figs 2D, 5A, D) with longer and more numerous setae; antenna 2 peduncular article 3 with a single or a pair of smaller robust setae; peduncular article 4 with 1–3 ventromedial small robust setae, with two ventrodistal processes; peduncular article 5 without robust seta, with ventroproximal and ventrodistal process, size of process function to maturity of the specimen.

***Variability*** (based on specimens from Bône, Arcachon Bay, and Trébeurden; same data as material examined).

***Head*** with rostrum reaching or not lateral ridge of head; ***antenna 1*** peduncular article 1 with one or two dorsomedial and two to four ventral robust setae, sometimes no left right symmetry; antenna 1 flagellum with five or six articles; ***female antenna 2*** peduncular article 4 with two to four robust setae, and few times in larger specimens a distal pair; ***male antenna 2*** peduncular article 4 with one to three medioventral robust setae, ventrodistal process subequal or the upper one slightly shorter; ***maxilliped*** article 2 outer margin with one to three plumose setae distally; ***gnathopod 1*** coxa ventral margin with one to three long plumose setae; ***gnathopod 2*** dactylus with generally two teeth on flexor margin for adult specimens (specimen with BL < 2.2 mm mainly juveniles with only one tooth, 1.7% of adults specimens examined with three teeth), sometimes no left right symmetry, an adult male specimen (BL = 2.46 mm) with two teeth on the left and a single tooth on the right gnathopod 2; ***pereopod 3 and 4*** dactylus reaching between proximal to distal end of carpus; ***pereopod 5 and 6*** carpus with clusters of three or four proximal and three to five distal robust setae; urosome with or without small lateral depression which looks like a vestigial notch; ***uropod 1*** peduncle with five to nine robust setae along outer margin, sometimes with one to three proximal simple setae, rami with three to five robust setae on outer margin; ***uropod 3*** peduncle shorter to subequal in length to ramus, variously expanded, with or without setae dorsally; ***telson*** more or less acute, dorsodistal robust setae tooth-like, mostly not observed.

###### Ecological data.

Thirty-eight brooding females were examined; BL ranged from 2.49 to 4.53 mm; fecundity ranged between 4 and 37 eggs/marsupium; mean fecundity of 9 eggs/marsupium. Eggs were ovoid, with mean major and minor diameters of 0.323 ± 0.055 mm and 0.266 ± 0.037 mm, respectively (*x̄* ± s; *n* = 351). These females were collected from two locations in Arcachon bay. In both locations, there were moderate but significant positive correlation between body length and number of eggs per marsupium (Spearman’s *ρ* =0.69 and 0.55, for locations “bouée 13” and “Arcachon harbor”, respectively). There was no significant correlation between the size of eggs and the number of eggs in the marsupium of females (Spearman correlation tests, *p*-values >0.05).

**Figure 6.**
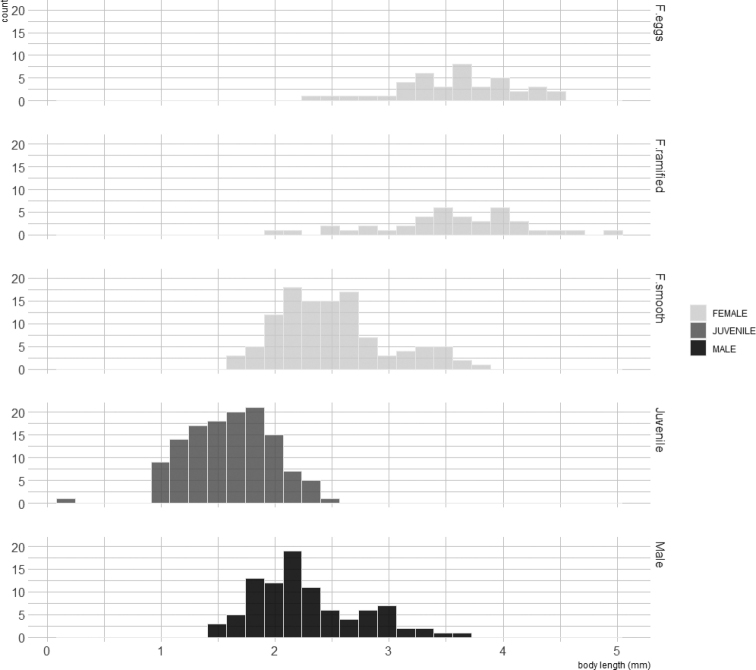
Frequency distribution of body size (in mm) for juvenile, male, female with smooth oostegites (F.smooth), female with ramified oostegites but no eggs (F.ramified), and female with eggs (F.eggs) of *Apocorophiumacutum* (Chevreux, 1908) in Arcachon bay, station “blockhaus”.

Field data collected in Arcachon Bay showed that gender features could be distinguished from a body length of c. 1.7 mm. Smaller female displayed smooth oostegites, while larger female displayed ramified oostegites with or without eggs (Fig. 6; Kruskal-Wallis test followed by post-hoc Dunn tests, *p*-values <0.05). Mature female with ramified oostegites or bearing eggs reached higher size than male (Fig. 6; Kruskal-Wallis test followed by post-hoc Dunn tests, *p*-values <0.05).

## ﻿Discussion

The genus *Apocorophium* was decribed by Bousfield and Hoover in 1997 during their revision of the family Corophiidae, with *Corophiumacutum* as the type species. Chevreux originally described *Apocophiumacutum* in 1908 with only female specimens from Bône, Algeria. Later, in 1925, Chevreux and Fage provided the first description of a male with specimens from the Lannion river mouth, Brittany, France. The species has been recorded in European waters (Mediterranean Sea and Atlantic coast), and also in the Suez Canal, Pakistan, New Zealand, South China Sea, and the Atlantic and Pacific coasts of America (Schellemberg 1928; Shoemaker 1934, 1947; Hurley 1954; Barnard 1969; Bousfield 1973; Dickinson et al. 1980; Bano and Kazmi 2012; Hossain and Hughes 2016). Even if most records and redescriptions are this species, some can be considered as doubtful due to inconsistencies (Table 1). This is the case for Korean specimens (Joung and Kim 2007) where some morphological characters differs from *A.acutum* (in parentheses): antenna 1 peduncular article 1 ventral margin bare in females and with only one distal robust seta in males (vs two to four in both sexes), female antenna 2 peduncular article 5 without robust setae (vs with one robust seta), and male antenna 2 peduncular article 4 without robust setae (vs with one to three robust setae). Specimens recently recorded from Uruguay (Demicheli and Verdi 2018) are also doubtful: urosome illustration represents lateral insertion of uropods, which infers *Monocorophium* and not *Apocorophium*. A reexamination of the specimens from Korea and Uruguay is, therefore, needed to confirm their identification, and molecular studies could be helpful for confirming identifications of species. Specimens from Bône, Brittany, and Arcachon Bay agree with the original and subsequent descriptions, apart from Korea and Uruguay specimens (see above). The only difference is related to the male antenna 2: Chevreux and Fage (1925) described and illustrated male specimens without any reference to robust setae on antenna 2 peduncular articles. Examination of specimens from Brittany, as well as information from other descriptions, reveals the presence of robust setae on peduncular articles 3 and 4. This was probably an oversight during the original description. Two characters that support the close similarity of *A.acutum* to species of *Hirayamaia* are the variously expanded uropod 3 peduncle and the presence of a lateral depression on the fused urosomite in some specimens (Fig. 5F), closed to a notch morphology. However, these two genera can be distinguished by the number of teeth on gnathopod 2 dactylus: two or three in *Apocorophium* and only one in *Hirayamaia* (Gouillieux and Sauriau 2019).

**Table 1.** Main morphological characters for *Apocorophiumacutum* (Chevreux, 1908) in original and subsequent descriptions.

References	Chevreux, 1908 – Original female description	Chevreux & Fage, 1925 – Original male description	Poisson & Legueux, 1926	Shoemaker, 1947	Hurley, 1954	Bousfield, 1973	Lincoln, 1979	Ruffo ed., 1989	Bousfield & Hoover, 1997 (after Bousfield, 1973)	Ren, 2006	Joung & Kim, 2007	Hossain & Hughes, 2016	Demicheli & Verdi, 2018	Present study
Area	Algeria	France, Monaco	France	East coast of America	New Zealand	New England	British Isles	Italia	Summarize	East China Sea	Korea	South China Sea	Uruguay	Algeria, France
Head rostrum	No data	Short, triangular, not reaching lateral ridge of head	NO DATA	Short, triangular	Short, , not reaching lateral ridge of head	Short, triangular, not reaching lateral ridge of head	Short, triangular, not reaching lateral ridge of head	Short, triangular, not reaching lateral ridge of head	Short, triangular, not reaching lateral ridge of head	Short, triangular, not reaching lateral ridge of head	Short, flattened, not reaching lateral ridge of head	Absent	Short, triangular, reaching lateral ridge of head	Short, triangular, reaching or not lateral ridge of head
Male antenna 1 article 1 robust setae ventral margin	No data	3	3	3	4	4	3	3	4	3	1	No data	No data	2–4
Male antenna 1 article 1 robust setae dorsomedial margin	No data	2	0	2	0	1	2	2	1	2	2	No data	No data	1–2
Male antenna 2 article 4 robust setae	No data	0	0	2–4	2	2–3	1–3	1–3	2–3	2	0	2	No data	1–3
Male antenna 2 article 5 process	No data	Medioventral and distoventral	Medioventral and no distal	Medioventral and distoventral	Medioventral and distoventral	Medioventral and distoventral	Medioventral and no distal	Medioventral and distoventral	Medioventral and distoventral	Medioventral and no distal	Medioventral and distoventral	Medioventral and distoventral	No data	Medioventral and distoventral
Female antenna 1 article 1 robust setae ventral margin	3	3	3	3	3–4	2–3	3–4	4–6	2	3 – 4	0	3	5	2–4
Female antenna 1 article 1 robust setae dorsomedial margin	0 ?	0 ?	0 ?	2	2–3	2	2–3	2	2	2	2	3	2	1–2
Female antenna 2 article 4 robust setae	3	3	3	3	3	3	4	6, some in pairs	3	3–5	3	3	3	2 – 4, rarely distal paired in larger specimens
Female antenna 2 article 5 distal process	Without	Without	Small	Small	Small	Small	Small	Without	Small	Without	Small	Small	Small	Small
Female antenna 2 article 5 ventral robust setae	1	1	0 ?	1	1	1	1	1–2	1	0–1	0	1	0 ?	1
Gn2 dactylus number of ventral teeth	No data	2	No data	2	3	2	No data	2	2	2	2	2	2	2–3
Uropod 1 insertion	Ventral	Ventral	Ventral	Ventral	Ventral	Ventral	Ventral	Ventral	Ventral	Ventral	Ventral	Ventral	Lateral	Ventral

Bousfield and Hoover (1997) provided a revision on the family Corophiidae for species belonging to the tribe Corophiini. They described many new genera and proposed a key, based on type species, to world genera, which has been largely used by all taxonomists,. However, some mistakes have been noted by Gouillieux and Sauriau (2019) for species characterized by having urosome segments fused with uropod 1 arising mainly ventrally; they suggested that *Hirayamaiatridentia* (Hirayama, 1986) should be changed to *Apocorophiumtridentia* and a partially revised key was proposed.

### ﻿*Apocorophium* identification key

Based on the original descriptions of *Apocorophium* species, the authors proposed a world key to adults of *Apocorophium* species (*Hirayamaiatridentia* is herein mentioned as *Apocorophiumtridentia*; female of *A.louisianum* was not included in the present key due to lack of description).

**Table:** 

1	Female specimen	**2**
–	Male specimen	**6**
2	Female antenna 2 peduncular article 4 with distal process	**3**
–	Female antenna 2 peduncular article 4 without distal process	**4**
3	Female antenna 1 peduncular article 1 with 1 ventral and without dorsomedial robust setae; antenna 2 peduncular article 4 without robust setae; pereopods 3 and 4 dactylus shorter than propodus and carpus combined	***A.lacustre* (Vanhöffen, 1911)** ^1^
–	Female antenna 1 peduncular article 1 with 2 ventral and without dorsomedial robust setae; antenna 2 peduncular article 4 with 1 robust seta; pereopods 3 and 4 dactylus longer than propodus and carpus combined	***A.simile* (Shoemaker, 1934)**
4	Female antenna 2 peduncular article 4 with pairs of robust setae	***A.tridentia* (Hirayama, 1986)**
–	Female antenna 2 peduncular article 4 with row of single robust setae (can be a distal pair for larger specimens)	**5**
5	Female antenna 2 peduncular article 4 with 4 robust setae on ventral margin and 6 robust setae on dorsomedial margin	***A.curumim* Valério-Berardo & Thiago de Souza, 2009**
–	Female antenna 2 peduncular article 4 with 2 to 4 robust setae on ventral margin, without robust setae on dorsomedial margin	***A.acutum* (Chevreux, 1908)**
6	Male antenna 1 peduncular article 1 with proximomedial tubercule; antenna 2 peduncular article 5 without median process	***A.louisianum* (Shoemaker, 1934)**
–	Male antenna 1 peduncular article 1 without proximomedial tubercule; antenna 2 peduncular article 5 with median process	**7**
7	Male antenna 2 peduncular article 4 with robust setae on inner face	**8**
–	Male antenna 2 peduncular article 4 without robust setae on inner face	**9**
8	Male antenna 1 peduncular segment 1 with 2 ventral and no dorsomedial robust setae; antenna 2 peduncular segment 5 without distal process	***A.simile* (Shoemaker, 1934)**
–	Male antenna 1 peduncular segment 1 with 2 to 4 ventral and 1 or 2 dorsomedial robust setae; antenna 2 peduncular segment 5 with distal process	***A.acutum* (Chevreux, 1908)**
9	Male antenna 1 peduncular article 1 with 2 robust setae on dorsomedial margin; rostrum papillate	***A.tridentia* (Hirayama, 1986)**
–	Male antenna 1 peduncular article 1 with 0 or 1 robust seta on dorsomedial margin; rostrum triangular	**10**
10	Male antenna 1 peduncular article 1 without robust setae on dorsomedial margin; uropod 3 rami shorter than peduncle	***A.lacustre* (Vanhöffen, 1911)**
–	Male antenna 1 peduncular article 1 with 1 robust seta on dorsomedial margin; uropod 3 rami subequal to peduncle	***A.curumim* Valério-Berardo & Thiago de Souza, 2009**

### ﻿Ecological notes

*Apocorophiumacutum* is a tube-dwelling amphipod living subtidally to 360 m, but usually between 0 and 5 m, in brackish water, in channels, on open coasts, in estuaries, and in harbors. It occurs on sponges, algae, roots of *Laminaria* J.V. Lamouroux, 1813, ascidians, hydrozoa, with coralline, oysters and *Sabellaria* Lamarck, 1818 reef, in the fouling of man-made installations (buoys, pilings, floating pontoons) (Chevreux 1908; Chevreux and Fage 1925; Poisson and Legueux 1926; Salfi 1939; Bousfield 1973; Myers 1982; Barnard and Karaman 1991; Bousfield and Hoover 1997; present paper).

Ovigerous females have been recorded in December in Suez Canal (Schellenberg 1928), between June and September in New England (USA) (Bousfield 1973); in February, March, May, July to November in British waters (Crawford 1937), and in February in Trébeurden. In Arcachon Bay (present study), examination of specimens showed presence of ovigerous females in April, May, August, October to February, and sexual maturity is reached over 1.7 mm in males and over 2.2 mm in females.

## ﻿Conclusion

The concept of cosmopolitan species is increasingly questioned. Such species are often found to be species with incomplete, early descriptions. Their redescription highlights the presence of new species, often supported by genetic analyses. The redescription of *Apocorophiumacutum* was necessary in order to avoid misidentification. The molecular description of specimens from the French coast is considered to be identical to that of the type locality due to morphological similarities, but a species complex cannot be excluded until specimens from Bônes have been sequenced.

## Supplementary Material

XML Treatment for Apocorophium acutum

## Footnotes

based on original description and redescription from Bousfield 1973.
